# Meniscal Regeneration: A Cause of Persisting Pain following Total Knee Arthroplasty

**DOI:** 10.1155/2011/761726

**Published:** 2011-09-20

**Authors:** Hosam E. Matar, Benan Dala-Ali, Henry D. Atkinson

**Affiliations:** ^1^Department of Trauma and Orthopaedic Surgery, North Middlesex University Hospital, Sterling Way, London N18 1QX, UK; ^2^Department of Trauma and Orthopaedic Surgery and North London Sports Orthopaedics, North Middlesex University Hospital, Sterling Way, London N18 1QX, UK

## Abstract

Many patients have persisting knee pain following total knee arthroplasty. We report the unusual case of a patient whose chronic lateral and medial knee pain were caused by entrapped regenerated meniscal tissue. This was diagnosed and successfully treated by arthroscopic debridement.

## 1. Introduction

Knee osteoarthritis is a leading cause of functional disability in adults [1, 2]. Total knee arthroplasty (TKA) is an effective treatment shown to dramatically decrease pain and improve function in appropriately selected patients [3]; however, many patients have persistent knee pain following TKA [4]. The aetiologies of chronic pain following TKA are poorly understood [5]; however, postoperative infection, prosthetic malfunction, component loosening, and surgical experience have been proposed as contributing factors [6]. We present an unusual case of a patient with chronic knee pain following TKA caused by entrapped regenerated meniscal tissue diagnosed and successfully treated by arthroscopic debridement.

## 2. Case Presentation 

A 70-year-old man presented one year following a right TKA performed for knee osteoarthritis. His surgery had taken place at another hospital, and he had made an initially uneventful recovery with a good clinical range of motion and satisfactory postoperative radiographs. At 9 months, however, he began to develop medial and lateral retinacular and deep knee pain, without associated knee swelling, warmth, or wound disturbance. His symptoms steadily worsened, particularly with load-bearing activity and flexion past 80 degrees. 

**Figure 1:**
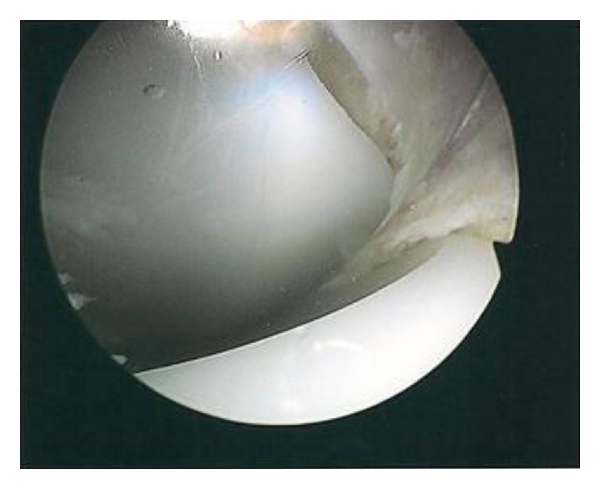
Intra-operative arthroscopic images showing soft-tissue entrapment in the: anteromedial compartment.

**Figure 2:**
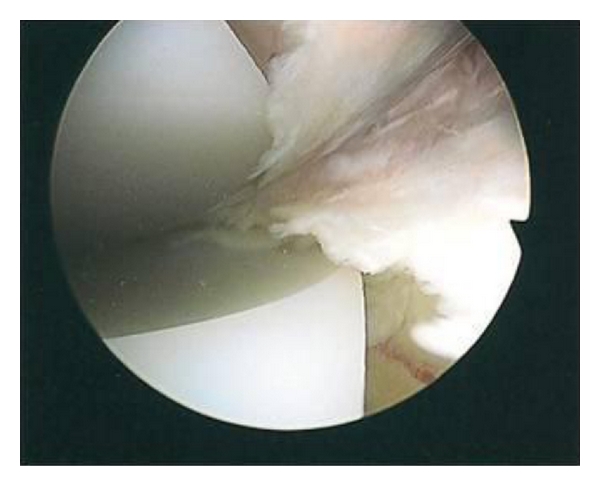
Intra-operative arthroscopic images showing soft-tissue entrapment in the: medial compartment.

**Figure 3:**
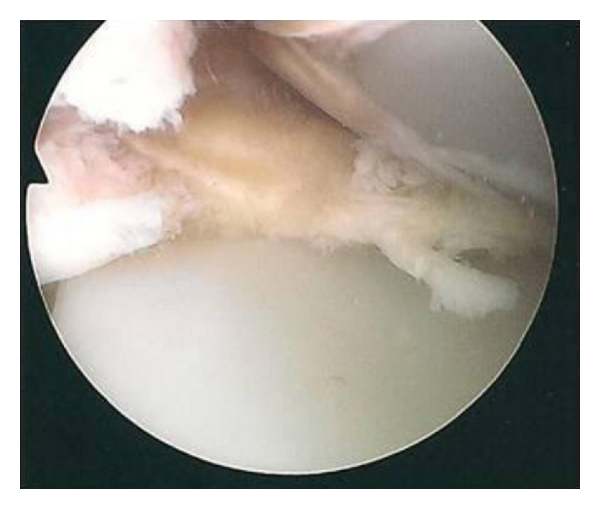
Intra-operative arthroscopic images showing soft-tissue entrapment in the: lateral compartment.

**Figure 4:**
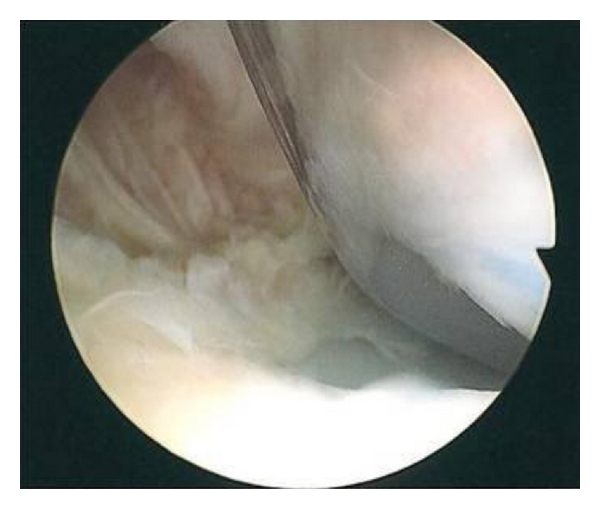
Intra-operative arthroscopic images showing soft-tissue entrapment in the: lateral compartment.

**Figure 5:**
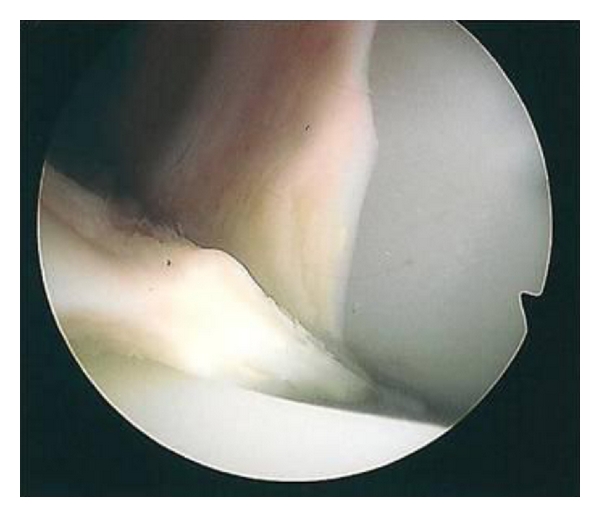
Intra-operative arthroscopic images showing soft-tissue entrapment in the: lateral compartment in deep flexion.

On examination there was no effusion or warmth present in the knee, and the wound was quiescent. There was generalised medial and lateral retinacular and joint-line tenderness. The knee was stable in flexion and extension with an active range of motion of 0–80 degrees. Passive range of motion was 0–105 degrees. Motion was limited by medial and posterior pain. Other lower limb examination was normal. Blood inflammatory markers were all within the normal limits, repeat radiographs revealed no evidence of component loosening, and a three-phase bone scan found no increased activity in either the dynamic or blood pool images. A TC 99m leucocyte cell scan was also negative. The patient underwent exploratory knee arthroscopy. There was no effusion present or evidence of synovitis, and there was no significant capsular scarring/contracture. The components were intact, without evidence of particulate matter or surface scratching, and there was no evident osteolysis at the bone/implant interfaces. Interposed in both the medial and lateral compartments and extending posteriorly between the femoral component and the polyethylene spacer were ridges of meniscus-like tissue (Figures 1, 2, 3, 4 and 5). These were resected using a chondrotome and samples sent for analysis. 

Histopathology revealed meniscus-like tissue with fibr-ocartilaginous cells in a scanty matrix surrounded by collagen wavy bundles without significant polyethylene particulate debris. Microbiological analysis found no evidence of infection. 

The patient made an uneventful recovery and had a full resolution of his preoperative symptoms within 6 weeks. He remains clinically well, mobile, and with a 0–105 degree range of motion at 6 months after arthroscopy.

## 3. Discussion

Persistent knee pain after TKA can have a wide spectrum of aetiologies. It is useful to categorise the potential causes into extra-articular and intra-articular. Extra-articular aetiologies are common comorbidities of patients, which include hip osteoarthritis, neurological disorders (lumbar radiculopathy, spinal stenosis, complex regional pain syndrome), vascular disorders (claudication), the Pellagrini-Stieda syndrome, and ligamentopathy (epicondylitis) as well as others. Intra-articular causes include infection, malalignment, aseptic loosening, osteolysis, extensor mechanism problems (patella fracture or maltracking), and soft-tissue impingement (meniscus-like growth) as well as others. 

As our case has illustrated arthroscopy can be a useful diagnostic and therapeutic tool in the management of the problematic TKA, particularly for patients in whom radiological and laboratory findings have not been conclusive [7–9]. 

Our patient was found to have soft-tissue entrapment between the femoral component and the polyethylene tray, and histological analyses of the resected interposed pseudomeniscus were suggestive of meniscus-like tissue. Meniscal regeneration has been previously described in experimental and clinical studies following meniscectomy [10, 11], and has also been previously reported following TKA [12, 13]. It has been suggested from histological studies that this regeneration arises when fibrocytes migrate from the synovium into the joint and differentiate into chondrocyte [14, 15]. The regenerated tissue consists of loosely dispersed collagen fibres with a lower-than-normal proteoglycan content, similar to the findings in our patient [16]. Also experimental studies have found that exposing tendons to compressive forces led to the development of new fibrocartilaginous tissue as result of cellular response of mesenchymal derived cells to the application of compressive forces [17]. 

There is only a single other documented case of a TKA patient with a symptomatic pseudomeniscus being successfully treated through arthroscopic tissue resection [12]; however, this technique can be equally employed in patients with chronic pain relating to retained or partially resected menisci following TKA [9, 18]. 

## 4. Conclusion

Entrapped regenerated meniscal tissue is an unusual cause of chronic knee pain following TKA and can be diagnosed and successfully treated by arthroscopic debridement. It is essential to ensure that the menisci are completely resected during the operation to prevent this phenomenon.

## Abbreviations

TKA: Total knee arthroplasty.
